# A156 FECAL PROTEOLYTIC ACTIVITY IS ASSOCIATED WITH SYMPTOMATIC STATUS IN CROHN’S DISEASE

**DOI:** 10.1093/jcag/gwae059.156

**Published:** 2025-02-10

**Authors:** A Hann, M Bording-Jorgensen, M Constante, P Moayyedi, M G Surette, H Galipeau, E Verdu

**Affiliations:** McMaster University, Hamilton, ON, Canada; McMaster University, Hamilton, ON, Canada; McMaster University, Hamilton, ON, Canada; McMaster University, Hamilton, ON, Canada; McMaster University, Hamilton, ON, Canada; McMaster University, Hamilton, ON, Canada; McMaster University, Hamilton, ON, Canada

## Abstract

**Background:**

An imbalance in host proteolytic activity (PA) has been reported in patients with inflammatory bowel disease (IBD) versus healthy controls (HCs). Fecal PA increases before the diagnosis of ulcerative colitis (UC) which is proinflammatory and has a microbial component. High microbial PA is also present in Crohn’s disease (CD), and when transferred to germ-free mice, induces inflammation through protease receptor-2 signaling. However, whether high PA associates with symptoms in IBD is unknown.

**Aims:**

To investigate whether patients with IBD and high PA have higher symptoms as assessed by Symptom IBD Short Index (SIBDSI).

**Methods:**

Patients with IBD and HCs were recruited and followed for >4 years in the MAGIC study via the IMAGINESPOR network (www.imaginespor.com). Ethnicity, sex, and age information were collected at the initial visit and stool donations were collected at baseline and at each yearly visit. IBD patients completed the SIBDSI survey at each fecal donation and were classified as asymptomatic (UC <13, CD <14) or symptomatic (UC >13, CD >14). From the larger cohort, 60 HC, 60 CD patients, and 60 UC patients were selected and fecal samples from baseline and 1-year follow up visits were used for proteolytic activity assays. Total proteolytic (trypsin), elastase-like (N-Suc-Ala3-pNA), and mucolytic (bioassay) activities were measured in the feces at baseline and follow up from IBD patients and HCs.

**Results:**

Demographic information, SIBDSI scores, and fecal donations from 60 HCs (39F/22M), 60 CD (33F/24M), and 60 UC (33F/25M) patients were analyzed from baseline and 1-year follow up. The average age for HCs, CD patients, and UC patients was 55, 51, and 45, respectively, and most patients were Caucasian. Overall proteolytic activity and mucolytic activities were (p=0.0001) in IBD patients compared with HCs. From baseline to follow up, 27 CD and 24 UC patients had a change in their symptom status classification. In symptomatic CD patients compared with asymptomatic CD patients, total fecal PA was higher (p=0.04).

**Conclusions:**

Dysregulated PA is emerging as a potential mediator of inflammation in some patients with IBD. Our data suggest that high overall proteolytic activity may be linked with symptomatic status in a subset of CD patients. PA activity may constitute a novel non-invasive biomarker for monitoring CD disease activity that could therapeutically be targeted in patients with proteolytic-driven disease.

**Funding Agencies:**

CCC, CIHR

